# Litigation claims following laparoscopic and open inguinal hernia repairs

**DOI:** 10.1007/s10029-020-02173-y

**Published:** 2020-03-30

**Authors:** R. Varley, C. Lo, B. Alkhaffaf

**Affiliations:** 1 Department of General Surgery, Salford Royal Foundation Trust, Salford, Manchester, M6 8HD UK; 2 Division of Cancer Sciences, Faculty of Biology, Medicine and Health, University of Manchester, Manchester, UK; 3 Core Surgical Training Programme, Health Education England, North West (East Sector), Manchester, UK

**Keywords:** Hernia, Inguinal hernia, Litigation, Clinical negligence, Laparoscopic hernia

## Abstract

**Purpose:**

Groin hernia repair is the most frequently performed general surgical operation in the UK. Complications from laparoscopic and open repair are well recognised; however, potential differences are yet to be considered in relation to litigation.

**Methods:**

Administrative data were obtained and analysed from the NHS Litigation Authority for inguinal hernia-related claims from 1995 to 2016. Claims identified as using an open or laparoscopic approach were compared.

**Results:**

880 claims were made, 760 had been settled. 88 laparoscopic and 241 open procedures were identified; 65% laparoscopic and 63% open hernia claims were found to be in favour of the claimant. Payouts totalled to 4.1GBP/4.8EUR/5.3USD million and 9.4GBP/11.0EUR/12.1USD million for laparoscopic (mean 82,824GBP/96,579EUR/106,453USD) and open (mean 66,796GBP/77,892EUR/85,852USD) approaches, respectively. The most common reasons for claim initiation were visceral/vascular injury (54%) in the laparoscopic group, and testicular complications or chronic pain (35%) in the open group. Additional procedures were necessary for 48% and 44% of laparoscopic and open claims, respectively. The highest average payouts were associated with visceral injury, (laparoscopic 116,482GBP/135,820EUR/149,715USD; open 199,103GBP/232,246EUR/255,905USD) and vascular injury (laparoscopic 88,624GBP/103,369EUR/113,892USD; open 64,460GBP/75,163EUR/82,870USD). Additional procedures resulted in an average payout of 93,352GBP/108,917EUR/120,008USD (laparoscopic) and 60,408GBP/70,506EUR/77,657USD (open). The most common additional procedures were corrective visceral/vascular repairs, orchidectomy and recurrent hernia repair.

**Conclusions:**

The rate of litigation for clinical negligence in inguinal hernia surgery in the UK is increasing. Whilst there has been a recent increase in laparoscopic hernia repair claims, the volume and burden of claims related to open procedures remain greater.

## Introduction

In 2017/18 the annual ‘cost of harm’ to the National Health Service (NHS) was estimated to be 7-8GBP (8-9EUR/9-10USD) billion—a figure that has risen yearly along with the frequency of clinical negligence claims [1]. In England, clinical negligence falls under common law jurisdiction—a case-based legal system using precedents set by judges’ decisions on historic cases. However, approximately one third of all countries have judicial systems either wholly or partially derived from English common law including the USA, Commonwealth nations and much of Europe [2] and so much overlap exists.

Surgical specialties represent 40% of the clinical negligence claim volume, with general surgery accounting for 9% of all claims [1]. NHS England reported that 74,830 inguinal hernia repairs were carried out in 2017/18 [2]. Inguinal hernia repair (IHR) accounts for around 10% of the general surgical workload, with over one in four males expected to undergo an IHR during their lifetime [3]. For every 1700 IHR performed, there is approximately one clinical negligence claim and while this rate is low, the volume of surgery results in significant costs for the NHS [4].

Complications associated with IHR are well documented [3]. However, the frequency of complications suffered as a result of IHR are markedly different depending on whether an open or laparoscopic approach has been used and each approach is associated with its distinct profile of complications [5]. For example, both testicular injury and chronic pain are more commonly seen with an open approach whilst serious complications such as bowel/bladder/vascular injuries, although rare, are typically more common in laparoscopic surgery [6].

The costs of litigation are not only financial; the implications for the patient, surgeon, hospital, health service and wider community are far reaching. Adverse outcomes and complaints are inevitable but a greater understanding of the complications that result from clinical negligence claims could be invaluable to avoid future claims. So far no study has looked at the distribution of complications between laparoscopic and open IHR and the impact that this has on litigation. This study aims to identify the differences in litigation between laparoscopic and open IHR in England including associated costs.

## Materials and methods

The NHS Litigation Authority (NHSLA) is responsible for managing clinical negligence claims made against NHS trusts in England. In 2017 it became part of NHS resolution. Since April 2002, hospitals have obliged to report all claims to the NHSLA. Prior to this only ‘large’ payout claims had to be reported, the amount of which was left to the hospitals’ discretion.

The data collected by the NHSLA are administrative and as such the amount of clinical information contained is variable. NHSLA data include an incident description, categorisation of the injury sustained and cause of injury, outcome, costs and relevant dates. Data were obtained from the NHSLA to include all cases logged under the terms ‘hernia’, ‘inguinal hernia’, and ‘laparoscopic hernia’ between April 1995 and November 2016.

### Inclusion criteria and definitions

Cases not pertaining to inguinal hernia repair, paediatric cases, multiple pathology and inguinal hernia surgery combined with another procedure were excluded. The remaining cases were sorted by allegation of the causes (diagnostic error, delay in treatment, consent issues, anaesthetic complications, intra-operative error, delayed recognition of complication and sub-standard post-operative care), complication suffered (visceral injury, vascular injury, nerve injury, wound infection, haematoma, foreign body and wrong site operations) and finally the outcome of the complication (death, additional procedure, recurrence of hernia, chronic pain, testicular complication, sexual dysfunction). NHSLA classification and the incident description guided allegation categorisation.

The following assumptions were made about the data:

- documented ‘failure' is equal to recurrence;
- if not otherwise specified, an additional procedure was carried out in the following circumstances: bowel perforation where death did not occur, visceral/vascular injury, necrotizing fasciitis, and all cases of retained instrument or other foreign body.

Where claim descriptors were unclear, cases were categorised as ‘unspecified’.

### Data extraction and analysis

Two people independently interrogated all data and discrepancies were resolved by consensus. Cases were considered in terms of claim initiation and alleged complication suffered. The likelihood of claimant success was analysed in all settled cases and costs were evaluated. Where possible, cases were divided into laparoscopic and open procedures and these groups were compared. The Chi square test was used to analyse the relationships between categorical data.

Costs are stated in GBP and conversion to Euros and USD are based on XE currency converter (https://www.xe.com) accurate on the 24/11/2019.

## Results

### Overall

**Fig. 1:**
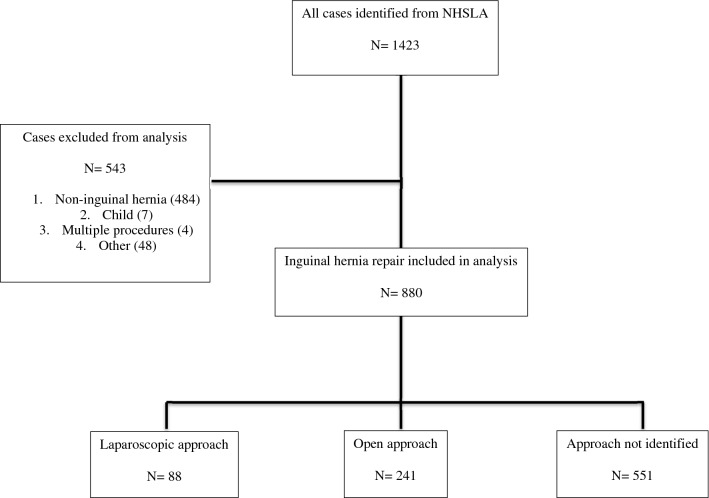
Cases included and excluded in the analysis

**Fig. 2:**
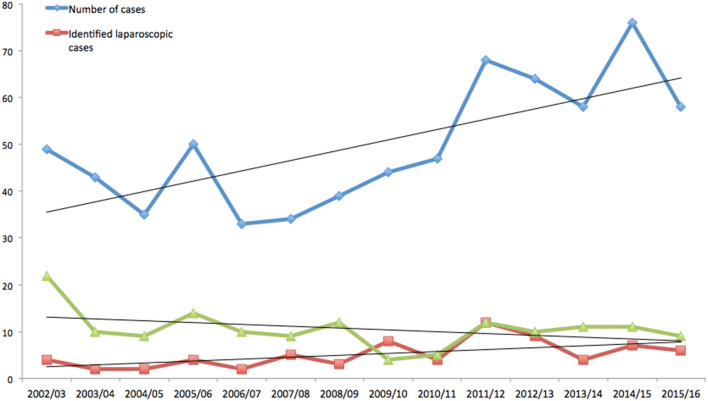
The frequency of litigation following inguinal hernia repair from April 2002 to April 2016 (diamond) with frequency of identified laparoscopic (square) and open (triangle) cases by year

880 cases relating to inguinal hernia repair identified between 1995 and 2016 were included in the analysis (Fig. 1). 760 were settled at the time of data collection with an overall success rate in favour of the claimant as 54.5% (414/760). The overall trend in litigation frequency since 2002 is illustrated in Fig. 2.

The total cost came to 33.2GBP/38.7EUR/42.7USD million with the following breakdown: GBP18.5/21.6EUR/23.8USD million (56%) damages to the claimant, 10.9GBP/12.7EUR/14.0USD million (33%) claimant costs and 3.8GBP/4.4EUR/4.9USD million (11%) defence costs. The mean cost for claimant success was 78,715GBP/91,871EUR/101,214USD. Unsuccessful cases resulted in an average of 2,211GBP/2,581EUR/2,843USD in defence costs—totalling 764,859GBP/892,834EUR/983,409USD.

The median time for claim initiation from the incident was 2.26 years, (interquartile range 1.96 years, range 0.0–30.2 years). 807/880 (91.7%) claims were initiated within 5 years of the alleged incident date. Sexual dysfunction (including infertility) (*P* = 0.024) and chronic pain (*P* = 0.009) were the only factors that occurred more frequently in claims initiated > 5 years post event. The most common cause for claim initiation was operative error, occurring in 73.2% of cases and the most frequently occurring complications included visceral injury (16.5%), testicular complication (15.6%), nerve injury (10.8%) and chronic pain (9%). 43.2% of cases involved an additional procedure.

The overall predictors of a successful claim included operative error (*P* = 0.01), visceral injury (*P* < 0.001), wrong site surgery (*P* = 0.011), death (*P* = 0.035) and the requirement of an additional procedure (*P* = 0.001). Chronic pain was the only significant predictor of an unsuccessful claim (*P* = 0.031).

### Laparoscopic vs. open repair

329 cases were identified from the data supplied by the NHSLA as being laparoscopic (88) or open (241) with 299 cases settled at the time of data collection (77 laparoscopic and 222 open). The frequency of identified laparoscopic and open cases by year is seen in Fig. 2. The likelihood of a successful claim in these groups was 64.9% and 63.1%, respectively with a mean total cost of 82,824GBP/96,579EUR/106,453USD and 66,796GBP/77,892EUR/85,852USD. Cost breakdowns are described in Table 1.

**Table 1:** The distribution of alleged complications in the laparoscopic and open groups with the likelihood of success and average cost of the settled claims

Complication/outcome	Frequency of complication/outcome (%), comparing laparoscopic vs open approach			Likelihood of success in settled cases (%) (*P* value)		Mean cost (GBP/EUR/USD)	
	Lap (*n* = 88)	Open (*n* = 241)	*P* value	Lap (*n* = 77)	Open (*n* = 222)	Lap (*n* = 77)	Open (*n* = 222)
Testicular complication	2 (2.3%)	48 (19.9%)	**0.0003**	100.0 (*P* = 0.317)	64.6 **(P = 0.043)**	51,753/60,371/66,501	42,806/49,934/55,004
Visceral injury	35 (39.8%)	17 (7.1%)	** < 0.0001**	70.0 **(P = 0.028)**	86.7 **(P = 0.005)**	116,482/135,820/149,715	199,103/232,246/255,905
Vascular injury	12 (13.6)	14 (5.8%)	**0.025**	80.0 (*P* = 0.058)	64.3 (*P* = 0.285)	88,624/103,369/113,892	64,460/75,163/82,870
Nerve injury	10 (11.4%)	34 (14.1%)	0.547	14.3 (*P* = 0.059)	67.6 **(P = 0.040)**	71,284/83,179/91,665	109,904/128,256/141,341
Wound infection	2 (2.3%)	35 (14.5%)	**0.003**	50.0 (*P* = 1.00)	46.9 (*P* = 0.724)	138,161/161,118/117,590	58,928/68,720/75,745
Haematoma	1 (1.1%)	5 (2.1%)	0.577	100.0 (*P* = 0.317)	33.3 (*P* = 0.564)	134,100/156,357/134,100	22,589/26,363/29,039
Wrong site surgery	1 (1.1%)	9 (3.7%)	0.232	100.0 (*P* = 0.317)	67.7 (*P* = 0.317)	35,418/41,335/45,532	11,686/13,637/15,023
Retained foreign body	2 (2.3%)	18 (7.5%)	0.091	100.0 (*P* = 0.157)	62.5 (*P* = 0.317)	24,877/29,015/31/962	17,419/20,316/22,383
Death	5 (5.7%)	15 (6.2%)	0.860	40.0 (*P* = 0.655)	76.9 (*P* = 0.052)	57,976/67,637/74,516	97,588/113,857/125,429
Additional procedure	42 (47.7%)	107 (44.4%)	0.654	72.7 **(P = 0.007)**	65.7 **(P = 0.002)**	93,352/108,917/120,008	60,408/70,506/77,657
Recurrence	2 (2.3%)	18 (7.5%)	0.091	100.0 (* P* = 0.317)	61.1 (* P* = 0.346)	53,263/62,164/62,164	45,497/53,100/58,497
Chronic pain	8 (9.1%)	36 (14.9%)	0.199	42.9 (*P* = 0.705)	54.3 (*P* = 0.612)	53,053/61,926/68,212	65,558/76,523/84,296
Sexual dysfunction	2 (2.3%)	9 (3.7%)	0.521	100.0 (*P* = 0.317)	88.9 **(P = 0.020)**	51,397/59,997/66,087	59,877/69,896/76,978

Significant *P* values are indicated in bold

### Causes of litigation

Operative error was found to be the cause for claim initiation in 76/88 (86.5%) of laparoscopic and 184/241 (76.3%) of open cases. Other features in the laparoscopic group included issues with the consent process (13/88, 14.8%) and delay in recognition of a complication (13/88, 14.8%). Diagnostic, anaesthetic and post-operative issues featured in less than 10% of all laparoscopic claims. In open cases, consent issues and problems with post-operative care each occurred in 28/241 (11.6%) of cases. In 20/241 cases (8.3%) there was a delay in recognition of the complication. Diagnostic and anaesthetic issues featured in less than 9% of open claims. There was no statistical significance in the distribution of these factors between the open and laparoscopic groups (*P* > 0.05 in all instances). The only causative factor found to significantly predict a successful claim was operative error, which was true for both the laparoscopic (*P* = 0.03) and open (*P* < 0.0001) groups.

### Complications and outcomes

Table 1 shows the distribution of alleged complications with the likelihood of claimant success and average cost of the settled claims, comparing laparoscopic and open groups.

Visceral injuries occurred in 52/329 (15.8%) of cases. A breakdown of visceral injuries is as follows: laparoscopic group (35)—bowel (27), bladder (6), other (2—fallopian tube injuries); open group (17)—bowel (16), bladder (1).

## Additional procedures

In total 380 claims involved an additional procedure, the most common additional procedures were corrective visceral or vascular repairs (108), orchidectomy (80) and recurrent hernia repair (64). The requirement of an additional procedure was predictive of a successful claim (*P* = 0.001). 39 claims were associated with multiple additional procedures [cases including repeated wound debridement (6), formation and reversal of stoma (4) and repeated recurrent hernia operations (6)]. Multiple procedures were independent factors for predicting a successful claim (*P* = 0.016) but were not more likely to be successful than any single additional procedure (*P* = 0.6).

**Fig. 3:**
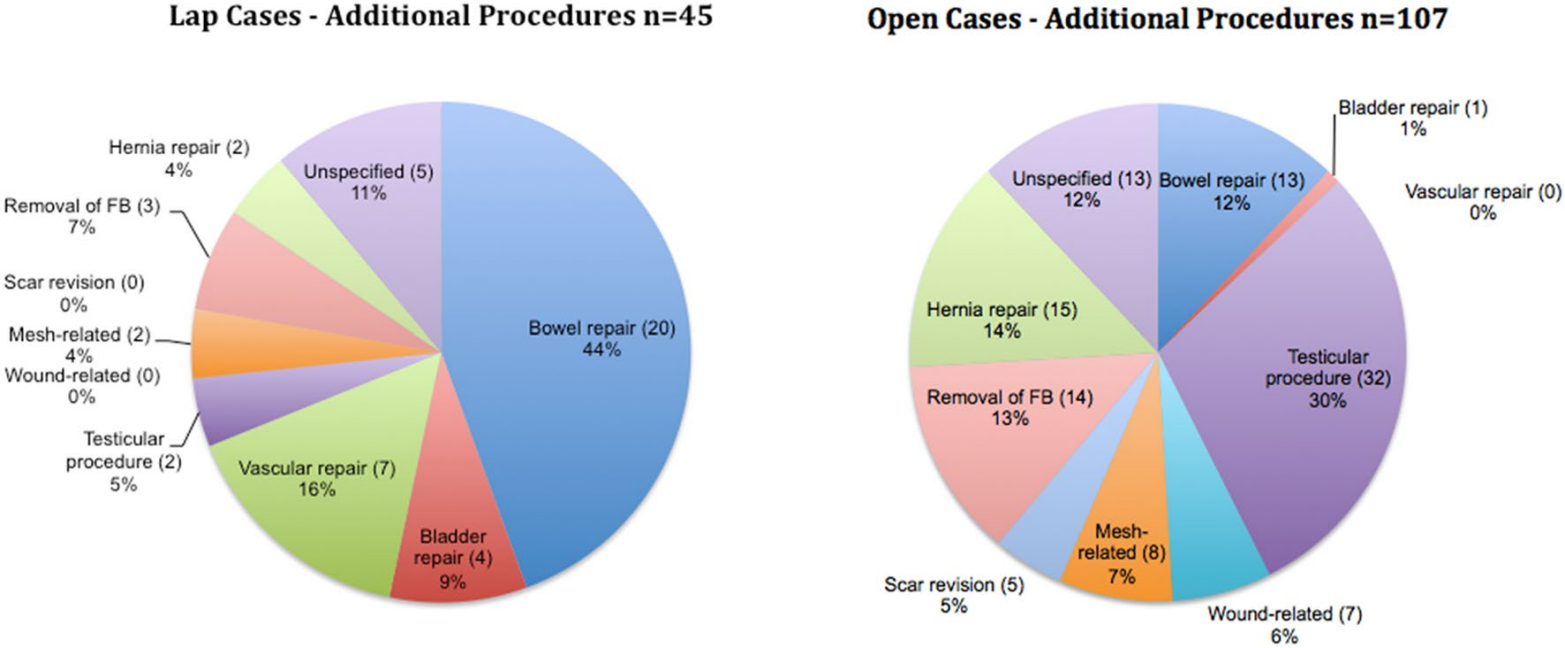
A breakdown of the primary additional procedure required in the laparoscopic and open groups

Additional procedures were associated with 42/88 (47.7%) laparoscopic and 107/241 (44.4%) of open claims, respectively. Figure 3 shows a comparison of the breakdown of the primary additional procedure required in the laparoscopic and open groups. The most common additional procedures in the laparoscopic group were visceral or vascular repairs (69%), while the most common additional procedures in the open group were orchidectomy (30%) and recurrent hernia repair (14%). Mesh complications were infrequent at 2% (laparoscopic) and 7% (open).

## Discussion

The UK has seen the overall litigation rates rise [1] and IHR is no exception. The mean payout in the laparoscopic group was higher than that of the open group, 82,824GBP/96,579EUR/106,453USD compared to 66,796GBP/77,892EUR/85,852USD. When compared to costs from other countries these costs are relatively high [7] with the mean payout from a Finnish study of IHR compensation being 2,260USD (1,758GBP/2,051EUR) [8].

The differences between laparoscopic and open groups could be due to the fact that the more serious complications (defined as bladder/bowel/vascular injury [9]) were associated with higher payouts and were seen more frequently in the laparoscopic group. These high-paying complications—visceral/vascular/nerve injuries and those requiring additional procedures—are an indication of the perceived magnitude of suffering in the patient. It is notable that these complications were seen to be predictive of a successful claim, except vascular injuries (where *P* = 0.058 in the laparoscopic group). However owing to the relatively small number of vascular injuries seen in the laparoscopic group, it is projected that the vascular injury may become a significant predictor as more cases are identified.

Although only 37.4% of cases could be reliably identified as having been laparoscopic or open owing to the non-clinical nature of the NHSLA database, it is likely that the groups are representative given that the distribution of complications between groups is as expected and in par with other studies [6, 8, 10]. The volume of unidentified cases makes it difficult to evaluate how the proportion of laparoscopic to open claims is changing over time as laparoscopic surgery increases in popularity, although the frequency of laparoscopic claims appears to be increasing. Despite a higher mean payout in the laparoscopic group, the overall volume of claims in the open group means the financial burden is greater for open claims. This is consistent with the most common operative approach being an open mesh repair [9].

Complications are an inescapable part of surgery. Whilst complaints happen, only a small proportion of these will result in litigation. It is worth noting that many clinical negligence claims are not the result of clinical error but can result from other factors, such as poor communication [11]. The reasons patients have for initiating a claim are not purely financial compensation but also include the need for an explanation, the wish to prevent similar future events and the desire for accountability [12, 13]. Considering all these factors is important for avoiding future litigation. Clinical negligence is considered to have occurred when a clinician breaches his duty of care to a patient and in doing so brings about a legally recognised harm. By definition, the surgeon’s practice must have deviated from the standard of ‘comparable professional practice’ (the Bolam test) [13]. However, the vast majority of the claims reported in this paper involve what could be considered as expected complications which have nonetheless led to claim initiation and legal action.

In 2015, the Montgomery ruling superseded the Bolam test specific to consent in the UK—now patients must have all risks and treatment options outlined in full, including the option of having ‘no intervention’ [14]. However, studies have shown that the quality of consent for IHR can be extremely variable and serious complications including visceral injury, testicular complications and chronic pain are frequently missed and this can be seen across all grades of surgeon [15, 16]. These particular complications make up a high proportion of claims with visceral injury and testicular complications significantly predicting a successful claim for both the laparoscopic and open groups, respectively. It has been argued that listing all complications will unduly alarm patients; however, it has been demonstrated that full disclosure of the risks of IHR does not increase patient anxiety and improves informed consent [17]. Standardised procedure-specific consent forms could help to eliminate these issues.

Even when complications have been discussed and documented, patient recalling and understanding can be affected [18]. Notably patients misunderstand the relative risks and benefits of laparoscopic versus open IHR even after counselling [19]. Furthermore, one study has shown that there are inconsistencies between documentation and what is actually discussed with the patient [20]. Consent should be an ongoing process and not a one-off event on the day of surgery. Continued communication, both before and after surgery could help increase understanding and thus reduce complaints. Studies are currently looking at the use of the internet and mobile applications in an effort to improve patient understanding [21, 22].

Consent issues aside, the most common cause for claim initiation in both groups was operative error with many cases resulting in additional procedures. While patient safety initiatives such as the WHO checklist have been successful in reducing errors such as wrong site surgery and retained foreign body, other issues are inherent to surgical training and are difficult to avoid (e.g. the learning curve). However knowledge of the complications likely to occur in open or laparoscopic approaches can promote the development of operative strategies to minimise complications.

It has also been suggested that the debilitating nature of certain complications (for example: further surgery, stomas, and infertility), is enough justification for patients to initiate a claim [4]. In Finland, proof of malpractice is not necessary for patients to receive compensation for injury as a result of surgery, avoiding complex legal proceedings in situations such as these while still allowing for compensation [8]. France, the Nordic countries and New Zealand have similar “no fault” systems for compensation [23, 24], a practice shown to be economically viable where appropriate safeguards are included [25]. Indeed, NHS resolution has developed an ‘Early Notification Scheme’ for brain injury at birth, aiming to facilitate quick and easy compensation and limit claimant legal expenses [1]. If this were adapted for surgical specialties it could allow patients to be compensated for foreseeable yet serious complications without excessive legal costs, whilst minimising the propagation of blame.

Complaints are often fuelled by a poor doctor–patient relationship which includes interactions taking place before and particularly after a complication has occurred and is exacerbated by the poor delivery of information [26]. Once complaints have been made, poor handling of the complaint increases the likelihood of the complaint proceeding to litigation [12]. NHS Resolution has incorporated this into its approach with a focus on early mediation to try to prevent cases proceeding to litigation with positive results so far [1]—it will be interesting to see the impact of this on hernia litigation in the future.

There are a number of limitations in this study, including the retrospective analysis of a non-clinical data set. A large number of cases were excluded due to a lack of clarity over the operative approach. Of the laparoscopic cases it was impossible to discern the technique used (e.g. preperitoneal vs extraperitoneal) which would affect the complications expected. In cases with multiple complications it is unclear which factor contributed most to the final outcome. Furthermore, it is not possible to link volume of cases to individual surgeons or unit. The European Hernia Society (EHS) recommended that hernia repairs should be carried out by hernia specialist surgeons working in accredited/certified hernia centres with a high case volume [27]. It would be interesting to see the impact of high-volume specialist surgery on litigation. However, this is the only data set of its kind and so provides an invaluable insight into clinical negligence claims, including the subjective patient reasons for claim initiation and the payout gives an indication of the magnitude of their suffering. The dataset could be improved in the future if a UK national hernia registry was introduced as is already in place in a number of countries including Denmark, Sweden, and Germany.

## Conclusion

Litigation claims following laparoscopic IHR appear to be increasing in number and are more costly per case compared to open surgery due to the severity of complications experienced. Open IHR still makes up for the majority of cases and should not be underestimated. Areas where improvements can be made include improving informed consent, reducing surgical risk and when complications have occurred, being open and honest, aiming for early resolution by ‘making things right again.’
